# Differences in Exposure to Minimally Invasive Surgery in a Sample of United States Obstetrics and Gynecology Residents

**DOI:** 10.7759/cureus.44480

**Published:** 2023-08-31

**Authors:** Sujatha Narayanamoorthy, Catherine Cepeda, Rodney McLaren, Amro Elfeky

**Affiliations:** 1 Obstetrics and Gynecology, Maimonides Medical Center, Brooklyn, USA; 2 Maternal and Fetal Medicine, Thomas Jefferson University Hospital, Philadelphia, USA; 3 Minimally Invasive Gynecology Surgery, Maimonides Medical Center, Brooklyn, USA

**Keywords:** simulation, robotic surgery, laparoscopy, obgyn residents, minimally invasive gynecologic surgery

## Abstract

Objective: The objective of this study was to assess the exposure to minimally invasive gynecologic surgery (MIGS) techniques among senior (third and fourth year) Obstetrics and Gynecology residents in the United States.

Methods: We conducted an online cross-sectional survey among senior residents who completed a 19-item questionnaire regarding their exposure to laparoscopic and robotic cases and techniques and their access to their simulation. We performed a comparison among these residents, grouped based on the four geographical regions of the United States.

Results: Senior residents, on average, performed 4.0 MIGS cases (standard deviation (SD) ±2.5), 1.0 two-handed laparoscopy (SD ±1.0), and 1.5 robotic cases (SD ±1.5) per week. The exposure to challenging skills such as extracorporeal and intracorporeal suturing and laparoendoscopic single site (LESS) surgery per week was minimal and did not vary across the nation (p=0.99, p=0.06, p=0.52, respectively). Access to dual consoles increased the number of robotic cases performed per week (p=0.01). While residents of all regions had equal access to laparoscopic box trainers (p=0.81) and laparoscopic simulators (p=0.22), residents of the southern region had less access to robotic simulators (p=0.04).

Conclusion: The number of MIGS cases performed by residents did not differ nationwide. However, exposure to advanced aspects of endoscopy training was minimal. The presence of a fellowship or type of teaching environment did not alter the number of cases performed by residents. Residents performed a greater number of robotic cases with the presence of dual consoles.

## Introduction

Minimally invasive gynecologic surgery (MIGS), including laparoscopic and robotic approaches, is a fundamental skill in the practice of gynecology. Resident skill development in this area is essential to achieving competence in gynecologic surgery. Contemporary gynecology practice involves a large number of conditions amenable to MIGS [1]. The American College of Obstetricians and Gynecologists (ACOG) and the American Association of Gynecologic Laparoscopists (AAGL) have stated that MIGS is currently the gold standard for performing hysterectomy, which is one of the most common surgeries performed on women [2,3].

Given the overall increasing trend towards MIGS and fewer open gynecology cases, the Accreditation Council for Graduate Medical Education (ACGME) increased the minimum graduation requirement in MIGS procedures for residents [4]. Data assessing the specific MIGS techniques taught to Obstetrics and Gynecology (OBGYN) residents across the United States are scant. While the ACGME has established minimum case volume requirements in specific categories for residents, these minimums are not to be construed as an indication of competency in each procedure. However, to ensure that residents are competent with MIGS procedures at the end of residency, more understanding of which minimally invasive techniques and skills are being taught and how they are being taught to residents is required. Thus, the main objective of this study was to assess the various MIGS techniques training that senior (third and fourth year) OBGYN residents are exposed to nationally. The preliminary analysis of this study was presented as an oral presentation in a virtual ACOG District II Meeting, New York, USA, on September 25, 2021.

## Materials and methods

A cross-sectional, online survey was developed to assess residents’ exposure and training in MIGS. This survey was emailed to all OBGYN training residency programs in the United States between May and June 2021. The population of the sampling frame, which consisted of senior residents currently enrolled in OBGYN residency programs, was requested to participate in the study. Senior OBGYN residents were defined as residents in either their third or fourth year of residency (postgraduate year (PGY) 3 and PGY4). This study was approved by the Institutional Review Board (IRB) of Maimonides Medical Center and ethics committee on October 12, 2020 (#2020-12-10-MMC).

Since a validated survey to assess senior OBGYN residents’ experience in MIGS does not exist, the authors created a survey consisting of 19 questions for the study (Supplementary Material). The survey consisted of questions on the demographics of residents, characteristics of their residency program, laparoscopy and robotic training experienced by residents in their home institution, and residents’ access to simulation. The MIGS cases consisted of laparoscopic and robotic gynecology cases.

Residents were categorized into four standardized regions that were established by the US Census Bureau: Northeast, South, Midwest, and West, based on the state location of their residency program. The survey was emailed to the program coordinators of all OBGYN programs, requesting them to forward the link to their third- and fourth-year residents. Each program received one email with the link to the survey. A request for a read receipt was obtained. Survey participants were self-selected by clicking the link to an online consent form. Survey instructions stated that responses were anonymous, participation was voluntary, and the survey would take approximately five minutes to complete. Responses to the survey were collected and stored using a secure web-based tool, Research Electronic Data Capture (REDCap).

Descriptive and univariate analyses were used to analyze the demographic characteristics of the residents. Following the Skewness and Kurtosis test to analyze for normality, the Kruskal-Wallis test was used to compare the number of MIGS cases and skills performed by residents across the four geographic regions and to evaluate if the number of robotic cases performed varied with the type of robotic console (single, dual, or having both single and dual) used. Wilcoxon rank test was used to evaluate whether the number of cases performed by residents differed with the type of teaching environment (community versus university-based program) and the presence of any OBGYN fellowship in a program. Fisher’s exact test was performed to analyze the access of residents from all four regions to simulation. All statistical analyses were performed with Stata/IC 16.1 (StataCorp LLC, College Station, TX).

## Results

A total of 6,070 OBGYN residents belonging to 286 residency programs were identified as the approximate number from FREIDA (The American Medical Association Residency and Fellowship database). Of this, approximately 3,035 were identified as senior residents (PGY3 and PGY4). Seventy-seven programs with 895 senior residents acknowledged the receipt of the email. Of those 895 senior residents, 225 (25.1%) completed the survey.

Demographics of the residents and characteristics of the residency program

The median age of residents was 33 years (interquartile range (IQR): 29-37). The majority of residents were female (N=197, 87.56%) and Caucasian (N=153, 68.92%). Responses obtained from PGY3 and PGY4 residents were 113 (50.22%) and 112 (49.78%), respectively. The majority of residents were from a university-based residency program (N=164, 72.89%). Most respondents were in the Northeast region (N=96, 42.67%). Of the 225 participants, 178 (79.11%) stated that they had fellowships in their program (Table 1). About half the residents (N=117, 52%) did not desire to pursue a fellowship.

**Table 1:** Demographics and characteristics of residents and residency program. Data expressed as median (IQR) or N (%). PGY: postgraduate year. *Northeast region included New Jersey, Pennsylvania, New York, Rhode Island, Massachusetts, Vermont, New Hampshire, Maine, and Connecticut. South region included Delaware, Maryland, District of Columbia, Virginia, West Virginia, Kentucky, North Carolina, Tennessee, Arkansas, Oklahoma, Texas, South Carolina, Georgia, Alabama, Mississippi, Louisiana, and Florida. Midwest region included Ohio, Michigan, Indiana, Illinois, Missouri, Iowa, Minnesota, Wisconsin, North Dakota, South Dakota, Nebraska, and Kansas. West region included Montana, Wyoming, Colorado, New Mexico, Arizona, Utah, Idaho, Nevada, California, Oregon, Washington, Alaska, and Hawaii.

Demographic characteristics	Number of participants (N=225)
Median age (years)	33 (29-37)
Gender	
Female	197 (87.56)
Male	27 (12)
Gender variant	1 (0.44)
Ethnicity	
Caucasian	153 (68)
African American	15 (6.67)
Asian	24 (10.67)
Hispanic	19 (8.44)
Pacific Islander	2 (0.89)
Other	9 (4)
Unknown	3 (1.33)
PGY level	
3	113 (50.22)
4	112 49.78)
Teaching environment	
University-based	164 (72.89)
Community-based	61 (27.11)
Geographic location*	
Northeast	96 (42.67)
South	47 (20.89)
Midwest	47 (20.89)
West	35 (15.56)
Presence of fellowship in program	
Yes	178 (79.11)
No	47 (20.89)
Robotic console in the training institution	
Single	23 (10.22)
Dual	105 (46.67)
Both	91 (40.44)
None	6 (2.67)

Characteristics of laparoscopy training

**Figure 1:**
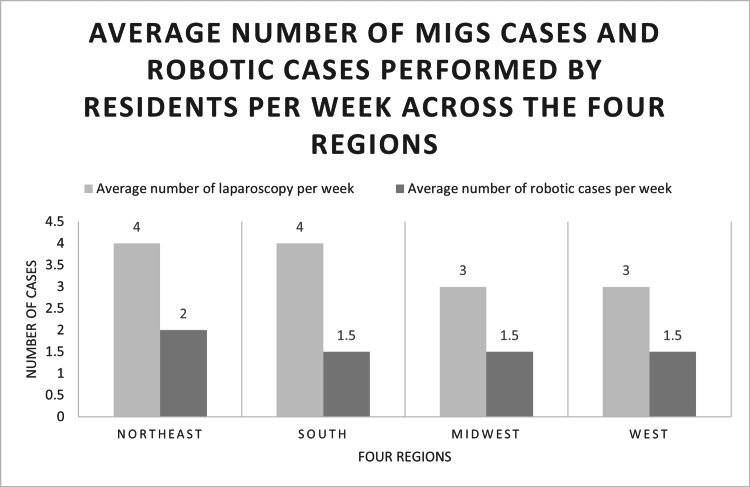
Average number of MIGS cases and robotic cases performed by residents per week across four regions. MIGS: minimally invasive gynecologic surgery.

The average number of MIGS cases performed by residents was noted to be 4.0 (standard deviation (SD) ±2.5) per week (Figure 1). The number of MIGS cases performed per week was uniform across the four regions of the United States (p=0.13). The number of MIGS cases performed per week did not differ with the presence or absence of fellowship (p=0.33) or the type of teaching hospital (p=0.12).

**Figure 2:**
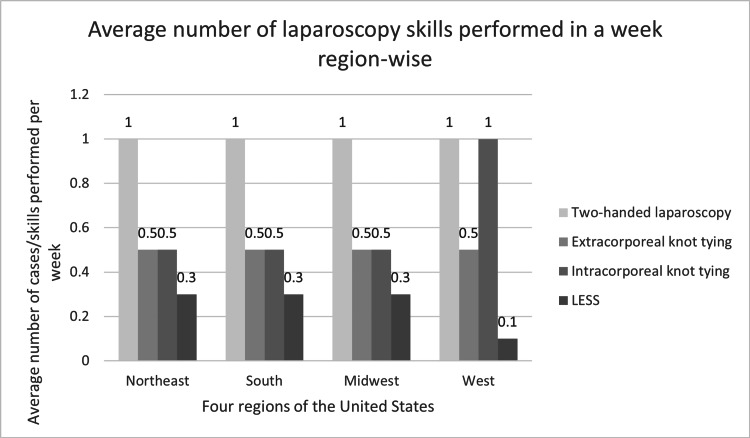
Average number of laparoscopy skills performed in a week region-wise. LESS: laparoendoscopic single site.

The average number of two-handed laparoscopic cases performed per week was 1.0 SD (±1.0), which also did not differ among the four regions (p=0.73) (Figure 2). The majority of the residents (N=225) stated that they performed zero cases of extracorporeal (N=200, 90%), intracorporeal (N=153, 68.9%) suturing, and laparoendoscopic single site (LESS) surgery (N=163, 72.4%) per week. The average number of cases incorporating extracorporeal suturing in each of the 4 regions was 0.5 per week (p=0.99). The average number of cases incorporating intracorporeal suturing did not differ nationwide (0.5 per week in Northeast, South, and Midwest regions and one case per week in the West, p=0.06). The average number of LESS cases performed per week also did not differ nationwide (0.3 per week in the Northeast, South, and Midwest regions and 0.1 per week in the West, p=0.52).

Characteristics of robotic surgery training

**Figure 3:**
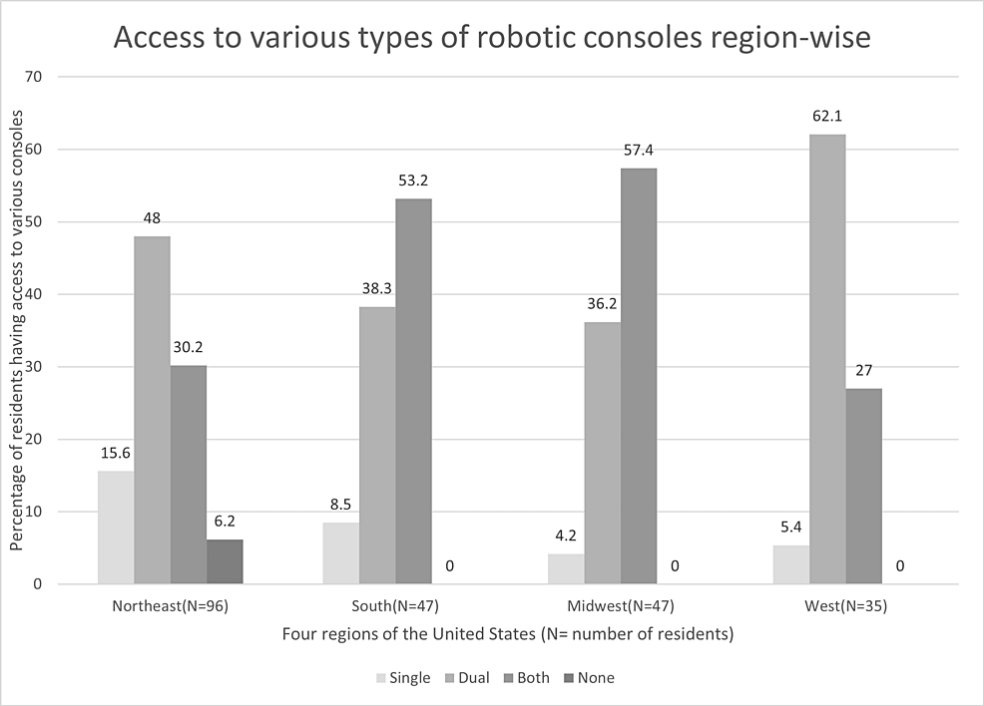
Access to various types of robotic consoles region-wise.

Only 3.5% (N=7) of the residents stated that they performed zero robotic cases per week. Senior residents performed an average of 1.5 (SD ±1.5) MIGS cases robotically per week with no difference among the four regions (p=0.87) (Figure 1). Of the 225 respondents, 6 (2.67%) had no access to robotic consoles, 23 (10.22%) had access to a single robotic console, 105 (46.67%) had access to a dual robotic console, and 91 (40.44%) had access to both single and dual consoles (p=0.003) (Figure 3). Residents who have access to dual consoles performed a greater number of robotic cases per week (p=0.01).

Simulation exposure

The distribution of access to the different types of simulators is presented in Table 2. Of the 225 residents, 200 (88.9%) had access to laparoscopic box trainers, 114 (50.7%) had access to a laparoscopic simulator, and 209 (92.9%) had access to a robotic simulator. Residents in the South region had the least access to robotic simulators (p=0.04).

**Table 2:** Access to simulation devices across US regions. Data represented as N (%).

Simulation types	Northeast (N=96)	South (N=47)	Midwest (N=47)	West (N=35)	p-value
Laparoscopic box trainer (N=200)	95 (98.9)	46 (97.8)	27 (57.4)	32 (91.6)	0.81
Laparoscopic simulator (N=114)	55 (57.2)	24 (51)	22 (46.8)	13 (37.1)	0.22
Robotic simulator (N=209)	88 (91.6)	41 (87.2)	46 (97.8)	34 (97.1)	0.04

## Discussion

We found that senior OBGYN residents in the United States performed a similar number of laparoscopic and robotic cases per week. Though the average number of MIGS cases performed in a week was 4.0, the exposure to challenging skills such as two-handed laparoscopy, extracorporeal and intracorporeal suturing, and LESS was minimal to none per week. The number of cases performed by residents did not differ based on the presence of fellowship in the program or the type of teaching hospital. While residents had equal access to laparoscopic box trainers and simulators nationally, residents in the South had less access to robotic simulators than residents in the other regions of the United States.

Providing exposure to advanced laparoscopic skills has been challenging, though specialty credentialing bodies state that residents need a working knowledge of more advanced laparoscopic techniques [5]. Recent studies have shown that many US OBGYN residents do not feel competent in performing advanced MIGS and that their surgical confidence decreases when surgical complexity increases [6,7]. Factors influencing the ability to teach residents advanced endoscopic skills may be a lack of operating room financial resources and time, trained faculty, attending interest, department support, and lack of patient demand [8]. Patient safety initiatives, consumer advocacy, financial restraints, decrease in funding, and declining resident work hours could also impact a program’s ability to offer quality endoscopic education.

A recent perspective stated that performing MIGS is becoming the standard of care among non-fellowship-trained OBGYN specialists, which was concerning to the program directors since they felt that their graduates were not fully prepared. The perspective also mentioned that only half of the programs met the ACGME requirement for MIGS training [9]. Our data support this concern since we found that exposure to specific training in MIGS techniques was minimal through all four regions of the United States. Thus, programs should evaluate ways of increasing exposure and teaching of advanced endoscopy.

This study found that the presence of dual robotic consoles increased the number of robotic cases the resident performed per week. Gynecology residents have expressed a marked interest in robotic surgery and predict that robotics will continue to increase in popularity [10]. Some advantages of robotic surgery include greater dexterity, improved articulation, three-dimensional magnification, enhanced accuracy and precision in complicated dissections, elimination of tremor, and favorable surgeon ergonomics. Some constraints and limitations of conventional laparoscopy may be overcome by the use of robotics [11,12]. The ACGME has recognized the need to assimilate training in robotics during residency through exposure to simulation and cases [4]. While residents from a survey conducted in 2011 stated that only 58% of residents had access to robotic surgical training in the form of robotic platforms, lectures, and video demonstrations [13], most of the residents in this study had access to robotic simulators, which is similar to the finding in a more recent study [14]. Dual consoles allow increased involvement and exposure to surgical cases for residents by enabling integrated teaching and surgical cooperation with proctoring and supervision without compromising operative times or patient outcomes [15].

This study has limitations. The low response rate may not be representative of all US senior OBGYN residents and thus may limit the generalizability of these results. In addition, this study may be underpowered to identify potential significant differences. However, there are only a minimal number of studies that have analyzed the MIGS cases and technique training that senior OBGYN residents are exposed to nationally. Thus, this data will help identify areas that residency programs should focus on to increase training and exposure to various MIGS skills. Selection bias inherent in the design of this study is unavoidable; however, we were able to sample both residents interested in pursuing fellowships and those who were not. We relied on the online survey answers to assess the training exposure of the residents, and we were not able to assess the confidence or ability of a senior resident to perform a MIGS case. However, it must be noted that confidence and ability can be assessed only if exposure to performing MIGS cases and techniques is adequate.

There are notable strengths in this study. There was a representative sample of senior residents from all four geographic regions of the country and different types of residency programs. We purposefully studied the number of cases performed by residents per week to prevent recall bias and to assess if these surgeries are being performed on a regular basis. Our study raises awareness of the need for more training in advanced techniques including two-handed laparoscopy and laparoscopic suturing skills that would be essential for the completion of procedures such as a myomectomy or a total laparoscopic hysterectomy. This would in turn increase the resident’s confidence and capability in performing gynecology surgeries, leading to enhanced quality of patient care.

## Conclusions

Exposure to the average number of MIGS cases was uniform across the regions of the United States among OBGYN residencies. However, exposure to advanced aspects of endoscopy training was minimal to none across the regions. The presence of fellowship or the type of teaching environment did not make a difference in the number of cases performed. The advanced aspect of the MIGS training in residency needs to be re-evaluated. Future studies partnering with the ACGME should be conducted to evaluate the MIGS curriculum and address ways to increase exposure to MIGS techniques and skills among residents.

## Appendices

1. Age

2. Which year of residency do you belong to?

o   PGY3

o   PGY4

3. Ethnicity

o   Caucasian

o   African

o   American Asian

o   Hispanic

o   Pacific Islander

o   Others

4. Gender

o   Male

o   Female

o   Others

5. US residency training

6. Desired subspecialty training (if any)

o   FMIGS

o   UroGyn

o   GynOnc

o   MFM

o   REI

o   Unsure

o   None

o   Other

7. Primary training environment

o   University-based hospital

o   Community-based hospital

8. Fellowships in your program (choose all that apply)

o   FMIGS

o   UroGyn

o   GynOnc

o   MFM

o   REI

o   Others

o   None

9. Number of residents in the program

10. On average, how many MIS cases do you perform in a WEEK:

11. On average, how many two-handed laparoscopic surgeries (two instruments, not including holding camera) do you perform in a WEEK?

12. On average, how many surgeries that include extracorporeal laparoscopic knot tying do you perform in a WEEK?

13. On average, how many surgeries that include intracorporeal laparoscopic knot tying do you perform in a WEEK?

14. Are you trained in single port/single incision laparoscopy? (See photo below)

o   Yes

o   No

15. Which robot console does your facility have? 

o   Single

o   Dual

o   Both

o   None

16. On average, how many cases of robotic surgery (including sitting on the console) do you perform in a WEEK?

17. Do you have access to a laparoscopic box trainer?

o   Yes

o   No

18. Do you have access to a computerized laparoscopic simulator in your facility?

o   Yes

o   No

19. Do you have access to a robot (Da Vinci or other) simulator?

o   Yes

o   No
